# Distribution of Protein Precipitation Capacity within Variable Proanthocyanidin Fingerprints

**DOI:** 10.3390/molecules25215002

**Published:** 2020-10-28

**Authors:** Milla Marleena Leppä, Juuso Erik Laitila, Juha-Pekka Salminen

**Affiliations:** Natural Chemistry Research Group, Department of Chemistry, University of Turku, Vatselankatu 2, FI-20014 Turku, Finland; juerlai@utu.fi (J.E.L.); j-p.salminen@utu.fi (J.-P.S.)

**Keywords:** bovine serum albumin, fractionation, mean degree of polymerization, proanthocyanidin, protein precipitation, tannin

## Abstract

Proanthocyanidins (PAs) are highly bioactive plant specialized metabolites. One of their most characteristic features is their ability to precipitate proteins. In this study, eleven plant species were used to study the structure–activity patterns between PAs and their protein precipitation capacity (PPC) with bovine serum albumin. To obtain a comprehensive selection of PAs with highly variable procyanidin to prodelphinidin ratios and mean degree of polymerizations, nearly 350 subfractions were produced from the eleven plant species by semi-preparative liquid chromatography. Their PA composition was defined by tandem mass spectrometry and high-resolution mass spectrometry, and their PPC was measured with a turbidimetry-based well-plate reader assay. The distribution of the PPC within plant species varied significantly. The mean degree of polymerization of the PAs had a strong correlation with the PPC (r = 0.79). The other structural features were significant from the PPC point of view as well, but they contributed to the PPC in different ways in different plant species. Retention time, prodelphinidin proportion, and mean degree of polymerization explained 64% of the measured variance of the PPC.

## 1. Introduction

Proanthocyanidins (PAs, a.k.a. condensed tannins) are one of the two main tannin groups, together with hydrolysable tannins, present in terrestrial plants. PAs possess multiple positive effects on human and animal health, e.g., anti-cancer [1,2] and anti-inflammatory [2,3] effects. Especially ruminant-related effects, such as lowered levels of gastro-intestinal nematodes [4,5] and decreased greenhouse gas emissions [6], could form a partial solution to a global increase in the drug-resistance of intestinal parasites [7], as well as climate change [7]. The mode of action either from the anthelmintic or antimethanogenic point of view has not been fully revealed, although certain structural features of PAs might play an important role in the activity. For instance, the high mean degree of polymerization (mDP) of PAs has been associated with strong anthelmintic activity [8], antimethanogenic activity [9], and also high protein precipitation capacity [10,11,12,13,14] (PPC). Therefore, studying the protein precipitation behavior of PAs might reveal their ruminant related bioactivities as well.

The molecular composition of PAs has a vast diversity of potential combinations of subunits (procyanidin, PC or prodelphinidin, PD), intra-molecular linkage types (A or B), and the lengths of the oligomeric or polymeric chain [15,16]. As a result of the aforementioned structural features, the PAs are a highly complex group of molecules, and individual plant species can produce up to hundreds of different PAs [17]. This diversity of different PA structures causes numerous challenges in PA purification, analysis, and structure–activity studies. Usually, in the structure–activity studies PAs have been separated from other plant metabolites, such as sugars [18], and fractionated into a few high and low mDP fractions [19,20]. Further fractionation of the PAs has not been routinely conducted, and thus, the more detailed information of the variation of PPC within single plant species has stayed unrevealed until now. 

PA–protein complex formation can occur via covalent [21,22] or non-covalent [23] reaction, and the complexes can take either soluble or insoluble form. The nature of the complex formation strongly depends on the conditions, the structure of the PA, and protein used in the complexation. At low to neutral pH, the complexes form via non-covalent interactions, such as hydrogen bonding [24,25], and hydrophobic interaction [21,26]. The covalent complexes are formed via oxidation of PAs prior complex formation [21]. For the PA–protein complexes to precipitate, the molar ratio of tannin to protein must be high enough. The precipitation takes place when PAs, which at the first reaction phase cover the surface of the protein, eventually form hydrogen bonds with each other and then result from a precipitate [27,28]. In this study, a turbidimetry-based method [29,30] was used to measure the haze formation between bovine serum albumin (BSA) and PA fractions, and therefore, only insoluble complexes were studied. The amount of the haze-formation was measured as the absorbance, and in this paper, the tendency to form the haze is referred to as PPC. 

In this study, as much as 25–35 individual PA fractions per each plant species were isolated to better understand the variation of PPC within single plant species and to reveal the structure–PPC linkages of PAs in more detail. The plant species were selected based on their PA fingerprints, which were studied earlier [17]. The reason behind the selection of certain plant species and tissues was purely to maximize the variability of the PA structures (PC or PD rich, galloylated PAs, different mDPs) used in this study. In total, nearly 350 purified PA fractions of 11 Finnish plant species (Supplementary Table S1) were analyzed. The refined PA fractions were produced with a semi-preparative reversed-phase chromatographic method [17], and the structural features of the PAs were analyzed via UPLC-MS/MS [31,32] and UPLC-HRMS [17]. Our approach combined the sophisticated purification of complex PA polymer mixtures [17] and high throughput PPC measurements [29]. The results revealed the distribution of PPC within the fractions produced from individual plant species. The PPC was also linked to the structural features of PAs, such as mDP, PD proportion (PD-%), and the estimation of the relative galloyl content (EG).

## 2. Results and Discussion

In the following chapters, the PPC results are discussed in terms of mDP, PD-%, EG, and retention time (t_R_).

### 2.1. The Effect of Polymer Size, Prodelphinidin Proportion and Retention Time on the Protein Precipitation Capacity

**Figure 1:**
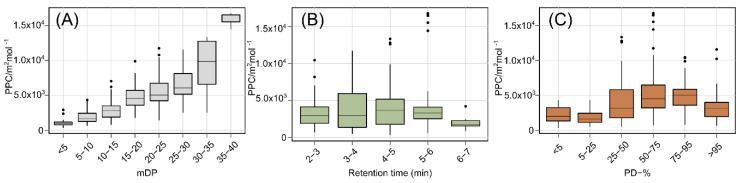
Protein precipitation capacity (PPC) as the molar absorption coefficient. The measured factors were (**A**) mean degree of polymerization (mDP), (**B**) retention time, and (**C**) prodelphinidin proportion (PD-%).

Figure 1 represents the boxplot figures of the changes in the PPC within different structural classes of PAs (mDP and PD-%) and retention time (t_R_) windows. The retention times of the semi-preparative fractions were obtained at the peak top times of the chromatograms at UPLC (*λ* = 280 nm). Even though, the retention time itself is not a structural feature, it is still greatly affected by the structural features of the PAs, such as PD-% [33]. Thus, it is one of the key variables in the study of PA fingerprints. The variables were categorized to produce the boxplot figures and to better estimate the distribution of the PPC within the variables (Figure 1).

The most significant change in the PPC was observed to increase in relation to the mDP (Figure 1A). The highest PPC was measured with fractions containing large polymers (mDP = 35–40, PPC_mean_ = 16.5 × 10^3^ m^2^ mol^−1^) and the lowest PPC was measured for fractions with low mDP (mDP < 5, PPC_mean_ = 1.0 × 10^3^ m^2^ mol^−1^). The variation in the PPC also increased in relation to the mDP except for the largest polymers (mDP = 35–40). Only *L. vulgaris* produced such high mDP fractions, thus the decrease in variation was caused by the homogeneity of the composition of these fractions. The PPC of the complete data set did not seem to follow any clear pattern in relation to the retention time or PD-% (Figure 1B,C). However, the lowest PPC values were measured for the fractions where the PAs eluted at late retention times (t_R_ = 6–7 min, PPC_mean_ = 1.8 × 10^3^ m^2^ mol^−1^, Figure 1B). On the other hand, regarding the PD-%, the highest PPC values were observed with relatively high PD-% (PD-% = 50–75%, PPC_mean_ = 4.5 × 10^3^ m^2^ mol^−1^; PD-% = 75–95%, PPC_mean_ = 5.0 × 10^3^ m^2^ mol^−1^, Figure 1C).

**Figure 2:**
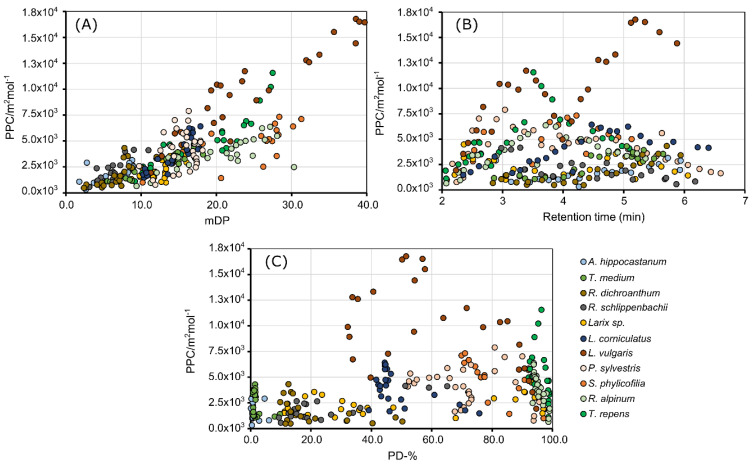
The correlation of protein precipitation capacity (PPC) as a function of the (**A**) mean degree of polymerization (mDP), (**B**) retention time of fractions, and (**C**) proportion of prodelphinidin (PD-%) of all studied plant species. Individual plant species are presented with different colors. The correlation between the complete data set was r = 0.79. The PPC scatter plots of individual plant species are presented in Supplementary Figures S1–S3.

Figure 2 presents the scatter plots of PPC of all plant species as a function of the mDP, t_R_, and PD-%. For a more detailed examination, separate figures of each plant species are illustrated in Supplementary Figures S1–S3. The PPC of the complete data set correlated linearly with the mDP (r = 0.79, Figure 2A), however distinct differences between plant species were evident. The correlations were linear and positive with all plant species and the correlation coefficients varied between 0.68 and 0.93, except for *P. sylvestris* (r = 0.27). For instance, *S. phylicifolia* (r = 0.73, Figure 2A, orange dots, Figure S1I) and *R. alpinum* (r = 0.77, Figure 2A, light green dots, Figure S1J) fractions gave lower PPC values, than *L. vulgaris* fractions (r = 0.93, Figure 2A, burgundy dots, Figure S1G), which also had the highest measured PPC values in this study. 

Figure 1 and Figure 2 showed that the PPC increased linearly as the mDP increased. A similar effect of oligomer or polymer size on the PPC has been observed in previous studies, thus our results were consistent with the literature [10,11,12,13,14]. The increase in the PPC might be due to simply more hydroxyl groups for the hydrogen bonding to take place, resulting in insoluble tannin−protein complexes [34]. The larger molecular size of the PAs enhances the probability of the cross-linkages between complexes inducing the formation of the precipitate [10].

In the complete data set, a similar linear correlation between the PPC and PD-% or t_R_ (Figure 2B,C) was not observed, as in the case of the mDP. Supplementary Figures S2 and S3 show the species-specific t_R_ and PD-% scatter plots of the PPC. The correlation between the PPC and PD-% was negative in the majority of the plant species. For instance, the most PD rich plant species in this study showed that the most PD rich fractions (PD-% > 98%) had the lowest PPC of the aforementioned plant species. Such plant species were *L. vulgaris* (Figure S2G), *S. phylicifolia* (Figure S2I), *R. alpinum* (Figure S2J), and *T. repens* (Figure S2K). Possible explanations for the low PPC of the most PD-rich fractions could be their relatively small mDP as compared to the rest of the fractions. For instance, the low PPC fractions (PPC < 2.0 × 10^3^ m^2^ mol^−1^) of *R. alpinum*, which were high in PD-% (PD-% > 98%, Figure S3I) were also low in mDP (mDP < 12, Figure S1I). The comparison of the mDP and PD-% revealed that in *S. phylicifolia*, *T. repens*, and *R. alpinum*, the mDP increased as the PD-% decreased (Supplementary Figure S4). Such cases, where the mDP and PD-% share a negative dependence, cannot be generalized, since it was more common for the PD-% to increase, while the mDP increased (Supplementary Figure S5). Especially in the highly PC or PD pure plant species, such as *A. hippocastanum*, *T. medium*, *R. alpinum* and *T. repens* the negative correlation of the PD-% and PPC should be considered as tentative result due to the relatively low variation of PD-%. 

In theory, the greater amount of the hydroxyl groups of the PD units compared to the PC units should increase the number of the hydrogen bonds between PAs and proteins, and thus, increase the complex formation and precipitation [34]. In practice, the increase in the PPC is much greater when the polymer size increases, compared to the increase in the hydroxylation level, thus the low mDP–high PD-% fractions of the aforementioned plant species were eventually low in PPC. 

The scatter plots of the t_R_ and PPC of the individual plant species were variable (Figure S2). In the PC rich plant species *A. hippocastanum* (Figure S2A), *T. medium* (Figure S2B), and *R. dichroanthum* (Figure S2C), the PPC increased as a function of retention time. In the PC “pure” species, such as *A. hippocastanum* and *T. medium*, the increase in the PPC was linear (Figures S2A and S2B) and the most active compounds eluted at late retention times (t_R_ > 5.0 min, Figures S2A and S2B,C). In multiple species, the PPC followed a second-order polynomial curve as a function of the retention time. This was observed especially in the most PD rich plant species *S. phylicifolia* (Figure S2I), *R. alpinum* (Figure S2J), and *T. repens* (Figure S2K), where the most active compounds eluted mainly at t_R_ = 3.0–5.0 min. The dependence between the PPC and retention time was linear and descending only in one plant species, *R. schlippenbachii,* where the most active compounds eluted early (t_R_ < 3.3 min, Figure S2D). 

Plant species, which contained a mixture of both PC and PD units, did not have a consistent distribution of the PPC in relation to retention time. For instance, there were one local, and one global PPC maximum in the t_R_–PPC scatter plots of *Larix* and *L. vulgaris* (Figure S2 panels E and G). In *L. corniculatus* and *S. phylicifolia* (Figure S2 panels F and I), the most active PAs eluted in the middle of the elution (*L. corniculatus,* t_R_ = 4.5–5.0 min and *S. phylicifolia* t_R_ = 3.8–4.2 min), and the PPC followed a down-ward facing polynomial curve as a function of the retention time in both species. In *P. sylvestris* (Figure S2H), the PPC followed a down-ward facing polynomial curve as a function of the retention time as well, but the most active PAs eluted earlier at t_R_ = 2.5–3.5 min. In total, there were two PPC maxima in the scatter plot of *P. sylvestris* as well.

At reverse-phase elution, the low polarity [33] of compounds, i.e., a low number of hydroxyl groups, and also large molecular size, increases the retention time. Therefore, the PC/PD content of the PA molecules affects their retention time amongst other structural factors, such as the three-dimensional structure of flavan-3-ol units and additional galloyl groups. [35] The distribution of the PPC as a function of the retention time was consistent in those plant species where the PA composition was relatively homogenous. In extremely PD-rich plant species *R. alpinum* and *T. repens*, the PPC followed a downward-facing second-order polynomial curve, whereas in the extremely PC rich plant species, the PPC followed a linearly ascending line. In the plant species with more heterogeneous PA composition, the distribution of PPC did not have a consistent feature in all of the plant species. 

The figure representing PPC in relation to the estimate of galloylation (EG) is shown in Supplementary Figure S6. The linear correlation between EG and PPC was fairly weak yet positive. The correlation coefficients in *R. schlippenbachii* and *R. dichroanthum* were 0.62 and 0.49, respectively. The additional aromatic D ring offers an additional phenolic ring to the complex formation [28] and also enhances the formation of cross-linkages. Therefore, the increase in PPC as the relative galloyl content increases fits the theory. In the following chapter, the factors influencing the PPC are examined in more detail with PLSR models.

### 2.2. Partial Least Square Regression of the Protein Precipitation Capacity of the Proanthocyanidins

**Figure 3:**
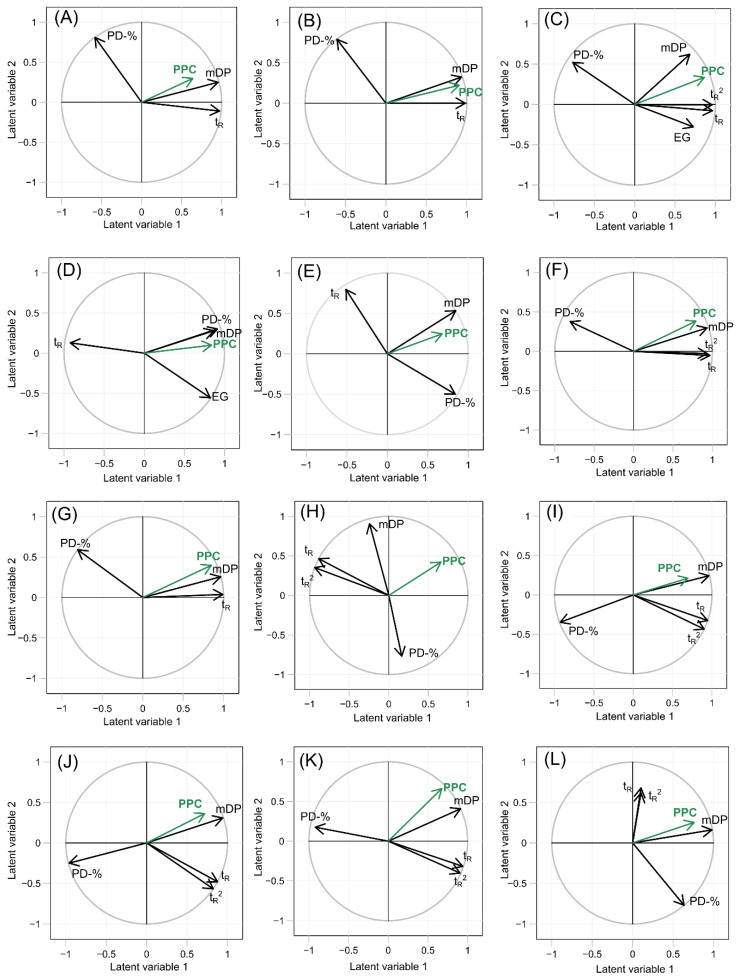
Partial least squares regression correlation circles of (**A**) *Aesculus hippocastanum*, (**B**) *Trifolium medium*, (**C**) *Rhododendron dichroanthum*, (**D**) *Rhododendron schlippenbachii*, (**E**) *Larix* sp., (**F**) *Lotus corniculatus*, (**G**) *Lysimachia vulgaris*, (**H**) *Pinus sylvestris*, (**I**) *Salix phylicofilia*, (**J**) *Ribes alpinum*, (**K**) *Trifolium repens*, and (**L**) all the plant species. The abbreviations are as follows: Protein precipitation capacity (PPC), the quadratic term of retention time (t_R_^2^), retention time (t_R_), mean degree of polymerization (mDP), prodelphinidin proportion (PD-%), and the estimation of the relative galloyl content (EG).

PPC was further tested with PLSR models. The retention time was included as a quadratic variable in those plant species, where the PPC formed a second-order polynomial curve as a function of the retention time. The results from the complete data set showed that the retention time (t_R_ and t_R_^2^), mDP, and PD-% of fractions explained up to 64.2% of the measured variation in the PPC. The cross-validated R^2^ (Q^2^ = 0.614) showed that the model was not over-fitted to the given data set. From the tested variables, the mDP was the most important one correlating strongly with the PPC (regression coefficient 0.799, Figure 3L, Table 1). The other variables were not significant for the PPC. The strong correlation between the mDP and PPC was in good accordance with the earlier results and the aforementioned literature. The PLSR models of individual plant species explained 49.9–89.2% of the measured variance of the PPC, and the cross-validated R^2^ values were close to the R^2^Y values (Q^2^ = 0.378–0.858), meaning that the plant-specific models were not over-fitted either. The variable specific regression coefficients, coefficients of determination (R^2^X and R^2^Y), and cross-validated R^2^ values (Q^2^) of the complete data set, and all plant species separately are presented in Table 1. The non-standardized regression coefficients are presented in Supplementary material (Table S2).

The PLSR correlations and regression coefficients showed certain similarities in such plant species, which contained similar PC/PD composition (Table 1, Figure 3). For instance, both extremely PD rich plant species, *R. alpinum* (PC/PD = 3/97) and *T. repens* (PC/PD = 2/98), had similar correlations for all variables, meaning that the same structural features were responsible for the PPC (Figure 3J,K, Table 1). In these plant species, an increase in the mDP increased the PPC, while an increase in the PD-% decreased the PPC (Figure 3J,K, Table 1). It seemed that the most active fractions in these plant species were the ones, which contained PC units to some extent (PD-% < 96%), even though the initial plant materials were PD-rich (Table S1) and there was relatively little variation in the PD-% between the fractions (Figure S3J,K). Altogether in *R. alpinum and T. repens*, the chemical interpretation of the PLSR models was that the most active PAs eluted with intermediate retention times where the PPC was caused by high mDP and the PAs contained relatively high proportion of PC units. However, the effect of PD-% on the PPC in *T. repens* was not significant, whereas in *R. alpinum*, it was.

The extremely PC rich plant species *A. hippocastanum* (PC/PD = 99/1) and *T. medium* (PC/PD = 99/1) (Figure 3A,B) also showed that the regression coefficients were similar for all variables meaning that the PPC of both PC rich plant species was caused by the same variables. Both mDP and t_R_ increased the PPC significantly (Table 1). Especially in the *T. medium*, the most active fractions (PPC > 2.8 × 10^3^ m^2^ mol^−1^) were relatively large in polymer size (mDP > 12, Figure S1B) and eluted late (t_R_ > 4.5 min, Figure S2B). Since both plant species were nearly PC pure, the variation of the PD-% was minor, and it did not have a major effect on the PPC (Table 1).

Unlike in the two previous cases, the PPC of the galloylated plant species *R. dichroanthum* and *R. schlippenbachii* (Figure 3C,E) was not consistently caused by the same features, even though their PC/PD-ratios were similar (Table S1). Systematically, only mDP increased the PPC in both plant species in a similar manner (regression coefficients of mDP of *R. dichroanthum;* 0.483 and *R. schlippenbachii;* 0.291, Table 1), whereas the PD-% and t_R_ showed an inverse trend with the PPC in the two plant species (Figure 3C,E, Table 1). Though the EG had an intermediate correlation with the PPC (Figure S6), the EG was not a significant predictor for the PPC (regression coefficients of EG of *R. schlippenbachii;* 0.085 and *R. dichroanthum;* −0.043) compared to other structural features of the PAs.

In the non-galloylated plant species with variable PC/PD-ratios (*Larix sp., L. corniculatus, L. vulgaris, P. sylvestris*, and *S. phylicifolia*, Table 1, Figure 3E–I), the only structural feature that was systematically responsible in a similar way to the PPC was the mDP. However, in some plant species, the mDP had a stronger correlation with the PPC (*L. corniculatus* and *L. vulgaris*, regression coefficients 0.926 and 0.754, respectively) than in other plant species (*Larix* sp., *P. sylvestris*, and *S. phylicifolia*, regression coefficients 0.650, 0.607, and 0.379, respectively). Interestingly, in the plant species with a strong correlation between the mDP and the PPC (*L. corniculatus*, R^2^ = 0.773 and *L. vulgaris*, R^2^ = 0.875), the PPC was better explained than in the plant species, where the correlation was low (*Larix* sp, R^2^ = 0.521, *P. sylvestris*, R^2^ = 0.611 and *S. phylicifolia*, R^2^ = 0.526). This could indicate that in *L. corniculatus* and *L. vulgaris* the mDP was the main structural PA feature behind the PPC, while in the other three species (*Larix* sp., *P. sylvestris*, and *S. phylicifolia*), there were possibly multiple important structural features, which the measured variables (e.g., retention time) did not fully explain. The retention time and PPC were related in different ways in these plant species with variable PC/PD-ratios, and no systematic connection could be established between the retention time and PPC. The relationships of the retention time, PPC and PA composition are discussed in more detail in the next chapter. 

Similarly, no systematic trend was observed between the PD-% and PPC. The PLSR results (Figure 3, Table 1) and the individual scatter plots (Figures S1–S3) indicated that the PPC was caused by similar structural features only in plant species, which were extremely rich in either PC or PD. In PC/PD mixtures, only the mDP had a systematically similar connection to the PPC, and no other generalizations could be made. All in all, in some plant species, the measured variables t_R_, t_R_^2^, mDP, PD-% and EG explained the variation of the PPC really well (*T. medium* R^2^ = 0.867, *R. dichroanthum* R^2^ = 856, *L. vulgaris*, R^2^ = 0.875, *T. repens* R^2^ = 0.892) whereas, in some plant species, the PPC was not as well explained (*A. hippocastanum* R^2^ = 0.499, *Larix* sp, R^2^ = 0.521, *P. sylvestris*, R^2^ = 0.611 and *S. phylicifolia*, R^2^ = 0.526). In these plant species, the PPC could have been caused by other structural features, such as interflavan linkage type, cis/trans ratio of the flavan-3-ol units, three-dimensional structure, or some other structural feature, which was not measured in this study.

### 2.3. The distribution of the Protein Precipitation Capacity within Proanthocyanidin Fingerprints

**Figure 4:**
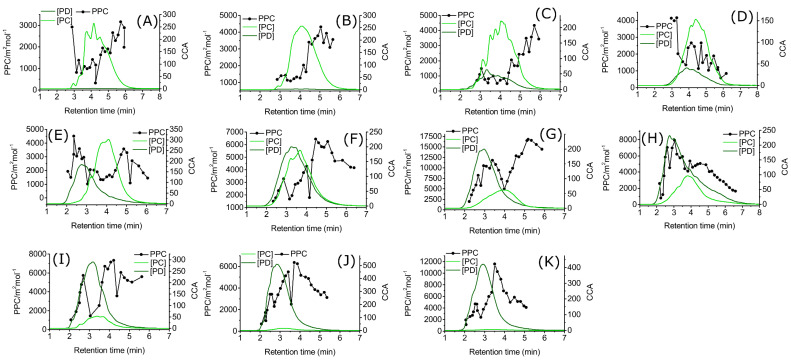
Multiple reaction monitoring fingerprints of proanthocyanidins with protein precipitation capacity results. Plant species are as follows: (**A**) *Aesculus hippocastanum*, (**B**) *Trifolium medium*, (**C**) *Rhododendron dichroanthum*, (**D**) *Rhododendron schlippenbachii*, (**E**) *Larix* sp, (**F**) *Lotus corniculatus*, (**G**) *Lysimachia vulgaris*, (**H**) *Pinus sylvestris*, (**I**) *Salix phylicofilia*, (**J**) *Ribes alpinum*, and (**K**) *Trifolium repens*. The abbreviations used are as follows: Protein precipitation capacity (PPC), concentration corrected abundance (CCA), calculated concentration of procyanidins ([PC]), calculated concentration of prodelphinidins ([PD]).

Figure 4 shows the PC/PD fingerprints [31,36] of all plant species with the PPC results. The dependence of the PPC on structural units of the PAs varied significantly within the plant species and four main distribution patterns observed are discussed below. The PPC increased at late retention times in *T. medium* (Figure 4B), *R. dichroanthum* (Figure 4C), and *L. vulgaris* (Figure 4G). Generally, the most active fractions in these plant species were the ones with late retention times (t_R_ > 4.5 min). For instance, in *T. medium* (Figure 4B), the PPC of the fractions nearly doubled at the descending part of the PA fingerprint (t_R_ > 4.5 min); and in *R. dichroanthum* (Figure 4C), the PPC of the fractions started increasing steadily, just after the PA concentration maximum (t_R_ > 4.4 min). The PPC in the *L. vulgaris* (Figure 4G) had one local and one global maximum at t_R_ = 3.0–3.5 min and t_R_ = 5.1–5.6 min, respectively. The first maximum was at the same retention time as the concentration maximum of the PA fingerprint, meaning that the most concentrated PA fractions were also relatively active ones. On the other hand, the most active fractions of *L. vulgaris* at t_R_ = 5.1–5.6 min were extremely low in concentration. Hence, the most active compounds in *L. vulgaris* were the very lastly eluting ones. Since these compounds were present only in minor compositions, they most likely contribute less to the plants’ total PPC compared to the less active, earlier eluting compounds. 

*R. schlippenbachii* (Figure 4D) was the only plant where the PPC decreased as a function of the retention time; hence, the galloylated plant species (Figure 4C,D) had completely opposite distribution of the PPC within their PA fingerprints. The most active compounds of *R. schlippenbachii* (Figure 4D) eluted at early retention times and unlike in the *R. dichroanthum* (Figure 4C) where the PPC of the fractions increased at the late retention time. Since both plant species were relatively similar in their mDP and PC/PD (Table S1), as well as PA fingerprints (Figure 4C,D), the variation of the distribution of the PPC most likely originates from some other structural feature which was not measured in this study.

*A. hippocastanum* (Figure 4A), *Larix* sp. (Figure 4F), and *S. phylicifolia* (Figure 4I) had the compounds with the highest PPC at the ascending and descending parts of the PA fingerprint, whereas the least active compounds were the most abundant ones. In *A. hippocastanum* (Figure 4A) the first fraction was very active, but already the second one was low in PPC, and the PPC increased rather steadily in the following fractions. The first maximum of the PPC in *Larix* sp. (Figure 4F) was approximately at the same retention time as the maximum PD concentration (t_R_ = 2.3–2.9 min), and the second PPC maximum was at the descending part (t_R_ = 4.5–5.6 min) of the complete PA fingerprint. In *S. phylicifolia* (Figure 4I) at the early elution, the PPC curve nearly followed the shape of the concentration of the PA fingerprint, and the PPC decreased significantly at the PA concentration maximum (t_R_ = 2.9–3.5 min). The PPC maximum was at the descending part of the PA fingerprint (t_R_ = 3.8–4.2 min), approximately 1.0 min after the PA concentration maximum. 

There was one more pattern where the PPC “followed” the shape of the PA fingerprint, and the PPC maximum was at the concentration maximum or shifted approximately 0.5 min to the latter retention time. Such plant species were *L. corniculatus* (Figure 4F), *P. sylvestris* (Figure 4H), *R. alpinum* (Figure 4J), and *T. repens* (Figure 4K). Interestingly, all of these plant species contained more PD than PC units (PD-% = 55%, 76%, 97%, and 98%, respectively). The highest PPC values of *R. alpinum* and *T. repens* (Figure 4K) were at the descending part of the PA fingerprint at t_R_ = 3.7 min and t_R_ = 3.5 min, respectively. This suggests that the most active compounds of *R. alpinum* and *T. repens* were not the most abundant ones, since the maximum responses of the PA fingerprints were approximately at t_R_ = 3.0 min. The PPC followed a similar pattern in *L. corniculatus* (Figure 4F), where the maximum of the PPC was at t_R_ = 4.6–5.1 min, and the maximum of the PA response was at t_R_ = 3.2–3.7 min. In both cases, the most active compounds were at the descending part of the PA fingerprint at late retention time. In the PA fingerprint of *P. sylvestris* (Figure 4H), the PPC pattern followed the shape of the PA fingerprint, suggesting that the most abundant compounds are also reliable for the PPC. 

In conclusion, the location of the maximum of the PPC in the PA fingerprint cannot be necessarily estimated based on the PA composition of a given plant species. In either PC- or PD-rich plant species, the PPC followed a consistent pattern, whereas in the more homogenous PA mixtures, the patterns of the distribution of the PPC were variable. In a couple of the plant species, the most abundant PAs were also high in PPC, whereas, in the majority of the species, the most active compounds eluted at the descending or ascending part of the PA fingerprint. In some species, both ascending and descending parts were active. For example, if the concentration maximum of the PA fingerprint is isolated and considered to contain the most active compounds of the PA mixture, the total PPC of the plant species could be significantly underestimated. These assumptions can mislead future structure–activity studies. This approach of closely examining the distribution of the bioactivity within PA fingerprint enables us to discover, which parts of the PA fingerprints are truly active and where to focus on in future studies. 

### 2.4. Fraction by Fraction Comparison with High-Resolution Mass Data

**Figure 5:**
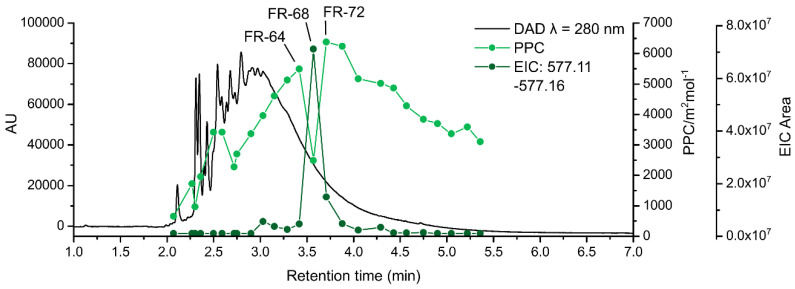
UPLC-DAD chromatogram (*λ* = 280 nm) of *Ribes alpinum* (black line). The protein precipitation capacity (light green dots and line) and the peak areas of extracted ion chromatograms of the dimeric B-type procyanidin (EIC at *m*/*z* 577.11–577.16, dark green dots and line) are presented on top of the chromatogram.

Interesting points in the t_R_–PPC scatter plots were examined more closely with high resolution mass spectrometric data. Figure 5 shows the distribution of PPC within a chromatographic hump (*λ* = 280 nm) of *R. alpinum.* The PPC decreased significantly at t_R_ = 3.4–3.8 min (fraction numbers 64, 68, and 72, Figure 5). The high-resolution mass spectra of the corresponding fractions 64, 68, and 72 (Figure S7) were interpreted to identify the possible causes for the dramatic decrease in the PPC. The PA oligomers and polymers in these fractions were mainly PD rich or PD pure compounds with a degree of polymerization ranging from two to nineteen. The most considerable difference within these three spectra was the presence of a dimeric B-type procyanidin (PC) in fraction 68, which also had the lowest PPC. An extracted ion chromatogram (EIC) of the dimeric PC (*m*/*z* = 577.11–577.16) was generated for all *R. alpinum* fractions resulting in the abundance of PC dimer per each fraction. Since the analyzed fractions were pure PAs, the desired EIC response was considered to originate from the dimeric PC only (*m/z* 577.13486, elemental composition C_30_O_12_H_26_, mass error −0.501 ppm). 

The decrease of the PPC in Figure 5 matched the elution pattern of PC dimer nearly perfectly; and at t_R_ = 3.4–3.8 min, they were practically mirror images of one another. It seemed that the presence of the small PC dimer explained the reduction in the PPC. The PA composition of *R. alpinum* consisted mainly of PD rich or pure polymers, and the mean polymer size was substantially higher compared to a dimer. More into detail, this finding showed how the small polymer size reduced the PPC of isolated PA fractions. This observation highlights the importance of the individual compounds within the complete PA mixture. Especially in a case like this, where the PC/PD composition of a plant is rather homogenous, a single oligomer with considerably different PC/PD ratio and oligomer or polymer size as compared to the other PAs, can distort the mean PPC significantly. These findings emphasize the importance of being able to characterize the PA composition of plants as accurately as possible to explain the causes of their PPC. 

## 3. Materials and Methods

### 3.1. Chemicals

The following chemicals were used in this study: acetone (extraction and Sephadex LH-20 fractionation: Analytical grade, VWR International S.A.S., Fontenay-sous-Bois, France), acetonitrile (semi-preparative purification: HPLC grade, VWR International S.A.S., EC; UPLC analyses: LC-MS grade, VWR International S.A.S., Radnor, PA, USA), methanol (analytical grade, VWR International S.A.S., Fontenay-sous-Bois, France), formic acid (semi-preparative purification: 99‒100%, VWR Chemicals, EC; UPLC analyses: LC-MS grade, Sigma Aldrich, St. Louis, MO, USA), Bovine Serum Albumin (BSA, Cohn Fraction V, lyophilized powder, purity ≥ 95%, Sigma Aldrich, St. Louis, MO, USA). Water was purified with Millipore Synergy UV (Merck KGaA, Darmstadt, Germany) system.

### 3.2. Plant Material

The same plant material was utilized in this study, as described by Leppä et al. [17]. Originally 39 PA-rich plant samples, including different types of plant tissues from 30 plant species, were collected and analyzed via UPLC-MS/MS. Eventually, leaves, flowers, needles, and pods of 11 PA-rich plant species were selected based on their PA fingerprints to maximize the structural variability (PC/PD, mDP, and EG) of PAs used in this study (Supplementary Table S1).

### 3.3. Proanthocyanidin Isolation and Fractionation

The plant material collection, extraction, and fractionation were performed, as described in the previous study [17]. The plant material was collected fresh into 1 L glass bottle, which was firstly filled with the plant material and secondly with acetone. The plant material was then macerated at +4 °C for 9–12 months and extracted with acetone/water, (4/1, *v*/*v*). To obtain a sufficient amount of extract, the collected plant materials were pooled from several individuals. Extracts were concentrated to water-phase and lyophilized. Dried extracts were pre-fractionated with Sephadex LH-20 column chromatography, which was utilized in a six-step fractionation protocol. Most of the PAs eluted with 4/1 acetone/water, (*v*/*v*). The PA-rich Sephadex fractions were concentrated to water-phase and lyophilized.

Sephadex LH-20 fractions were further purified by semi-preparative HPLC. Samples (125–150 mg) were eluted with acetonitrile and 0.1% aqueous formic acid at a flow rate of 12.0 mL min^–1^. Semi-preparative column (150 × 21.20 mm, Gemini^®^ 10 µm, C-18, 110A, Axia packed, Phenomenex, Torrance, CA, USA) was used, and the fractions were collected into 2 mL tubes from 5 to 33 min. In total, 168 fractions were collected per each plant species. Every fourth semi-preparative fraction was analyzed by UPLC-DAD-MS/MS [31,32] and UPLC-DAD-HRMS [17], as described in the following chapters. The fractions were chosen for analysis based on the specific retention time windows of each PA fingerprint, thus approximately 25–35 fractions were analyzed per each plant species. The PPC of all analyzed fractions was measured via turbidimetry-based well-plate reader assay.

### 3.4. UPLC-DAD-MS/MS Analyses

Tandem mass spectrometric (MS/MS) analyses were performed with a Xevo TQ triple quadrupole mass spectrometer (Waters Corp., Milford, MA, USA) coupled with an Aquity UPLC system (Waters Corp., Milford, MA, USA). The UPLC system consisted of a sample manager, a binary solvent manager, a column, and a diode array detector. The column used was a Waters Acquity UPLC BEH Phenyl (1.7 μm, 2.1 × 100 mm Waters Corp., Wexferd, Ireland). The elution was performed as described by Leppä et al. [17]. In the ionization source, the following parameters were utilized: Capillary voltage 2.4 kV, desolvation temperature 650 °C, source temperature 150 °C, desolvation and cone gas (N_2_) flow 1000 and 100 L h^–1^, respectively. Three cone voltages were used in the detection of PC (75, 85, and 140 V), and PD (55, 80, and 130 V) traces and the collision energy was set to 15 eV for PC, and 20 eV for PD. The UV (λ = 190–500 nm) and MS data were recorded from 0 to 8 min. The stability of the MS/MS response was monitored throughout the analysis [37] by injecting 1 μg mL^–1^ catechin solution (in acetonitrile/0.1% formic acid (2/8, *v*/*v*)) five times before and after every batch of 10 samples.

The quantitation of the PA subunits and galloyl units, as well as the determination of the mDP was done with the Engström method [31,32], as described by Malisch et al. [37]. The recorded PC and PD traces were smoothed (window size 5 scans × 2 smoothing iterations) and integrated with the TargetLynx software (V4.1 SCN876 SCN 917 © 2020 Waters Inc.). The integrated areas of the PC and PD traces were summed separately prior to quantitative calculations. The integrated areas were converted into quantitative data with the help of calibration curves made separately for galloyls, PC, PD, and mDP. Galloyls were quantified against a dilution series of 1,2,3,4,6-penta-*O*-galloylglucose ranging from 20 μg mL^−1^ to 39 ng mL^−1^ in acetonitrile/0.1% formic acid (3/7, *v*/*v*). The PC and PD calibration curves were obtained with two Sephadex LH-20 fractions: The PC standard from *Tilia* flowers (containing a known concentration of oligomeric and polymeric Pas in a 95/5 PC/PD ratio) and the PD standard from *Ribes nigrum* leaves (containing a known concentration of oligomeric and polymeric Pas in a 1/99 PC/PD ratio). Dilutions in acetonitrile/0.1% formic acid (2/8, v/v) were made between 1.50–0.1875 mg mL^−1^ for the PC standard and 2.00–0.25 mg mL^−1^ for the PD standard. [31] The mDP calibration curve was obtained with six Sephadex LH-20 fractions from *Vaccinium vitis-idaea* leaves, *Calluna vulgaris* flowers, and *Tilia* flowers, and the fractions had known mDPs of 2.2, 3.5, 3.6, 4.1, 6.0, and 9.9. The mDP calibration curve samples were made in acetonitrile/0.1% formic acid (2/8, *v*/*v*) and analyzed in 0.5 mg mL^–1^ concentration. The DAD traces at 280 nm were also integrated with TargetLynx software, and they were used to estimate the suitable sample concentration in the PPC assay.

The approximate galloyl content in relation to PA content was calculated based on the quantitative results obtained with the Engström method [31,32]. The quantitative results of the galloyl and PA traces were converted into molar concentration, and then their ratio was calculated. The aforementioned ratio of the galloylation degree is referred to as the estimation of the relative galloyl content (EG). The PA fingerprints were produced from the PC and PD traces by calculating the PA concentration in each time point with PC and PD calibration curves. This necessary concentration-based correction to the raw traces (concentration corrected abundance, CCA) was done to provide the visual presentation of the PC and PD as a function of the retention time. 

### 3.5. UPLC-DAD-HRMS Analyses

The high-resolution mass spectrometric analyses [17] were carried out with quadrupole–Orbitrap instrument (Q ExactiveTM, Thermo Fisher Scientific GmbH, Bremen, Germany), which was coupled with an Aquity UPLC system (Waters Corp., Milford, MA, USA). The UPLC system was similar to the above-mentioned system (UPLC-DAD-MS/MS analysis) except for the column, which in this case, was Acquity UPLC BEH Phenyl 1.7 μm, 2.1 × 30 mm. The flow rate was set to 0.65 mL min^–1^, and the same eluents were used as in the previous chapter (A, acetonitrile and B, formic acid/water (0.1/99.9, *v*/*v*)). The elution protocol started with a 0−0.1 min isocratic phase with 3% A in B, following by 0.1−3.0 min linear gradient with 3−45% A in B, and lastly, finishing with 3.0−4.2 min column wash and stabilization. The UV (*λ* = 190–500 nm) and mass (*m*/*z* = 200–3000, resolution = 70,000, automatic gain control = 3 × 10^6^) data were recorded from 0 to 4.2 min. A heated ESI source (H-ESI II, Thermo Fisher Scientific GmbH, Waltham, MA, USA) was operated in negative ion mode, and the source parameters were as follows: Spray voltage, −3.0 kV; capillary temperature, 380 °C; sheat, aux, and sweep gas (N_2_) flow rate, 60, 20, and 0 arbitrary units, respectively. The data were analyzed with Thermo Xcalibur (version 4.1) software. Qual Browser was utilized in spectral interpretation, and the extracted ion chromatograms (EIC) were generated via Quan Browser. The trace of the dimeric PC was detected at the *m*/*z* range of 577.11–577.16.

### 3.6. Protein Precipitation Capacity via Well-Plate Assay

The same fractions, which were analyzed by UPLC-MS/MS and UPLC-HRMS, were also analyzed via turbidimetry-based PPC measurement (Equation (1)), which was modified from the approach used by Engström et al. [29]. Firstly, 1400 μL of each fraction was moved into a fresh tube, concentrated, and freeze-dried. The freeze-dried samples were dissolved in water and shaken in a vortex mixer for a minimum of 5 min. The fractions were analyzed in 5 to 0.4–fold concentration (x_FOLD,_ Equation (2)) as compared to the original sample concentration (c_PA_, Equation (2)). The suitable concentrations for the turbidimetric assays were decided based on their earlier UV (*λ* = 280 nm) quantitation. The absorbance was measured via Multiskan Ascent (354, Thermo Electron Corporation Waltham, MA, USA) at 414 nm. Turbidimetry was measured as follows. Firstly, 75 µL of the fraction was moved into a 96–well plate. Secondly, 75 µL of 200 µM BSA solution in pH 5 buffer (0.05 M acetate supplemented with 60 μM ascorbic acid) was added to the wells, and the plate was shaken. The absorbance was read in 1 min intervals in a total of 31 times, and the maximum absorbance values were used in the calculations. Before every reading, the plate was shaken for 10 s to prevent the irregular accumulation of the haze. The measurements were carried out at a room temperature of 22 °C. 

Samples were measured in duplicates (rep1 and rep2 in Equation (1)), and a fraction with only pH 5 buffer was used as a reference (ref in Equation (1)) for each sample. These samples caused a slight absorption, due to their light yellowish color. The background absorption, due to the color, was subtracted from the average value as displayed in Equation (1).

ABS_sample_ = ((ABS_max,rep1_ + ABS_max,rep2_)/2) − ABS_max,ref_ (1)

The PPC (Equation (2)) was calculated from the total absorbance of the sample (ABS_sample_ in Equation (1)) by dividing the absorbance firstly with the concentration fold (x_fold_ = 5.0–0.4 in Equation (2)) and secondly with the result from the quantitation by UPLC-MS/MS, which also was the initial concentration of the samples (c_PA_, (mol L^−1^), Equation (2)). Additionally, the PPC values were divided by the length of the optical path (*l* = 4.7 mm at *V* = 150 μL in Equation (2)) at the well-plate well to display the final PPC values as the molar absorption coefficients (m^2^ mol^−1^).

PPC = (ABS_sample_/(x_fold_ × c_PA_ × l) (2)

Any sample with an absorbance value lower than 0.2 (n = 77 samples) or standard deviation higher than 10% (n = 11 samples) were discarded from the results to increase the reliability of the results.

### 3.7. Statistical Analyses

Statistical analyses were carried out with R (3.6.1) [38] in RStudio integrated development environment (version 1.2.5019) [39], and “*ggplot2*” package [40] was utilized for producing the graphs. The complete data set, and additionally, all 11 plant species were separately analyzed by Partial Least Squares Regression (PLSR) using the package “*plsdepo*” [41] in R. The mDP, PD-%, EG, and retention time (t_R_) were used as predictors to the PPC. The retention times of the PA fractions were determined as peak top time of the UPLC-DAD (*λ* = 280 nm, with 100 mm column) chromatograms. The variables were auto-scaled by dividing them with their standard deviation and by subtracting the mean from the variables. All models consisted of two latent variables, and the models were cross-validated. The effect of EG over PPC was only used as a predictor for galloylated PAs, which were present in *R. dichroanthum* and *R. schlippenbachii*. The aforementioned variables (mDP, PD-%, and t_R_) were categorized for producing the boxplot figures.

## 4. Conclusions

The results from this study strengthened the previous knowledge of the influence of PA polymer size on protein precipitation. However, the correlation between mDP and PPC varied amongst tested plant species, and for instance, *T. repens* (high correlation) and *P. Sylvestris* (low correlation) showed a major difference in correlation. The PLSR models showed that also other features of PAs were relevant (PD-%, t_R,_ t_R_^2^), but they contributed to PPC in different ways in different plant species. The comparison of PPC alongside PA fingerprints revealed how the PPC was distributed within tested plant species, which showed remarkably different PPC distribution patterns. For instance, in *P. sylvestris* the most abundant PAs were the most active ones, whereas in *L. corniculatus* the most active compounds were at the descending part of the PA fingerprint. Lastly, the importance of a single compound to the PPC was pointed out with the fraction-by-fraction comparison of *R. alpinum*. This highly refined set of PA fractions enabled the detailed conclusions about the PPC of variable PA fingerprints. Further studies utilizing a similar set of refined PA fractions and other types of proteins could offer more insights about protein precipitation behavior of PAs.

## Figures and Tables

**Table 1:** The results of the partial least squares regression models of all plant species and the complete data set, including all the samples.

Plant Species	Variable	Regression Coefficients	R^2^X	R^2^Y	Q^2^	Plant Species	Variable	Regression Coefficients	R^2^X	R^2^Y	Q^2^
*A. hippo-castanum*	t_R_	0.314	0.961	0.499	0.378	*L. vulgaris*	t_R_	0.385	0.980	0.875	0.854
	mDP	0.506	0.975				mDP	0.754	0.990		
	PD-%	0.255	0.995				PD-%	0.322	0.997		
*T. medium*	t_R_	0.399	0.961	0.867	0.857	*P. sylvestris*	t_R_^2^	−0.532	0.999	0.611	0.483
	mDP	0.577	0.979				t_R_	−0.408	0.999		
	PD-%	0.044	0.990				mDP	0.607	0.879		
*R. dichro-anthum*	t_R_^2^	0.365	0.920	0.856	0.829		PD-%	−0.323	0.615		
	t_R_	0.295	0.936			*S. phylicifolia*	t_R_^2^	−0.021	0.999	0.526	0.378
	mDP	0.483	0.851				t_R_	0.028	0.999		
	PD-%	0.091	0.861				mDP	0.379	0.984		
	EG	−0.043	0.605				PD-%	−0.347	0.981		
*R. schlippen-bachii*	t_R_	−0.243	0.866	0.711	0.594	*R. alpinum*	t_R_^2^	−0.147	0.999	0.646	0.496
	mDP	0.291	0.830				t_R_	−0.046	0.999		
	PD-%	0.320	0.921				mDP	0.457	0.979		
	EG	0.085	0.983				PD-%	−0.469	0.980		
*Larix sp.*	T_R_	−0.048	0.896	0.521	0.395	*T. repens*	t_R_^2^	−0.331	0.977	0.892	0.858
	mDP	0.650	1.000				t_R_	−0.157	0.980		
	PD-%	0.124	0.954				mDP	1.192	0.995		
*L. cornicula-tus*	t_R_^2^	0.041	0.874	0.773	0.644		PD-%	−0.038	0.875		
	t_R_	0.145	0.942			Comple-te data set	t_R_^2^	0.036	0.393	0.642	0.614
	mDP	0.926	0.957				t_R_	0.088	0.473		
	PD-%	0.311	0.789				mDP	0.799	0.986		
							PD-%	−0.056	0.996		

Abbreviations are as follows, coefficient of determination (R^2^X and R^2^Y), cross-validated R^2^Y (Q^2^), the quadratic term of retention time (t_R_^2^), retention time (t_R_), mean degree of polymerization (mDP), the proportion of prodelphinidin units (PD-%), the estimation of the relative galloyl content (EG).

## Supplementary Materials

The following are available online, Table S1: The information on the plant material utilized in this study. Figures S1–S3: Plant species-specific scatter plots of protein precipitation capacity as a function of the mean degree of polymerization (Figure S1), retention time (Figure S2), and proportion of prodelphinidins (Figure S3). Figure S4: Plant species-specific scatter plots of prodelphinidin proportion as a function of the mean degree of polymerization. Figure S5: The protein precipitation capacity of all samples as a function of the mean degree of polymerization. The proportion of prodelphinidin is illustrated with color gradient. Figure S6: Scatter plot figure of the protein precipitation capacity as a function of the estimation of the relative galloyl content. Figure S7: The comparison of the high-resolution mass spectra of three fractions (FR 64, 68, and 72) of *R. alpinum*. Excel file, including the raw data of the utilized fractions.
