# Supplementary material for: Distribution of Protein Precipitation Capacity within Variable Proanthocyanidin Fingerprints
Source: Molecules. 2020 Oct 28;25(21):5002. doi: 10.3390/molecules25215002 (PMC7663390; doi:10.3390/molecules25215002)
Supplement: Supplementary file 1 [file molecules-25-05002-s001.zip › Supplementary Tables and Figures.pdf]

Supplementary material for manuscript

Article

Distribution of protein precipitation capacity within variable proanthocyanidin fingerprints

Milla Marleena Leppä [1,*], Juuso Erik Laitila [1] and Juha-Pekka Salminen [1]

[1] Natural Chemistry Research Group, Department of Chemistry, University of Turku, Vatselankatu 2, FI-20014 Turku, Finland; mimale@utu.fi

***** Correspondence: mimale@utu.fi

Table S1. The plant species and tissues used in this study. The plant sources are similar as utilised in Leppä *et al.* [17].

Plant species		Semipreparative source material		
Latin name	Plant tissue	[a]mDP	[b]PC/PD ratio	[c]EG (mol/mol)
Aesculus hippocastanum L.	leaves	4	99/1	-
Trifolium medium L.	flowers	9	99/1	-
Rhododendron dichroanthum Diels.	leaves	5	79/21	1.0
Rhododendron schlippenbachii Maxim.	leaves	6	78/22	1.3
Larix sp	needles	12	62/38	-
Lotus corniculatus L.	green brownish pods	14	45/55	-
Lysimachia vulgaris L.	flowers	20	27/73	-
Pinus sylvestris L.	needles	18	24/76	-
Salix phylicifolia L.	leaves	20	15/85	-
Ribes alpinum L.	leaves	19	3/97	-
Trifolium repens L.	flowers	18	2/98	-

[b]Mean degree of polymerization (mDP) and [b]ratio of procyanidin and prodelphinidin subunits (PC/PD) measured by UPLC-MS/MS [31]. [c]The estimate of the relative galloyl content to proanthocyanidin content.

Table S1. The non-standardised Partial Least Squares Regression coefficients of each plant species.

Plant species	Variable	Non-standardized Regression coefficients	Plant species	Variable	Non-standardized Regression coefficients
A. hippo-castanum	Intercept	−615.81	*L. vulgaris*	Intercept	−8467.20
	t_R	262.16		t_R	1 489.18
	mDP	166.94		mDP	358.43
	PD-%	135.15		PD-%	60.38
T. medium	Intercept	−1 688.00	*P. sylvestris*	Intercept	3 059.66
	t_R	527.58		t_R^2	−78.51
	mDP	155.63		t_R	−521.90
	PD-%	22.49		mDP	512.87
R. dichro-anthum	Intercept	−2 468.60		PD-%	−43.16
	t_R^2	50.85	*S. phylicifolia*	Intercept	7 605.46
	t_R	359.30		t_R^2	−4.99
	mDP	233.20		t_R	50.58
	PD-%	8.49		mDP	109.31
	EG	−77.49		PD-%	−69.79
R. schlippen-bachii	Intercept	1 403.34	*R. alpinum*	Intercept	38432.80
	t_R	−266.09		t_R^2	−28.88
	mDP	172.89		t_R	−66.94
	PD-%	21.27		mDP	114.94
	EG	140.09		PD-%	−380.54
Larix sp.	Intercept	−1477.70	*T. repens*	Intercept	2 276.75
	t_R	−35.96		t_R^2	−122.33
	mDP	296.12		t_R	−413.05
	PD-%	3.43		mDP	510.33
L. corniculatus	Intercept	−7 837.30		PD-%	−37.49
	t_R^2	7.48			
	t_R	219.88			
	mDP	559.32			
	PD-%	50.40			

Abbreviations used: mean degree of polymerization (mDP), retention time (t_R), quadratic term of retention time (t_R^2), proportion of prodelphinidin (PD-%) and the estimated galloyl content in relation to proanthocyanidin content (EG, mol/mol).

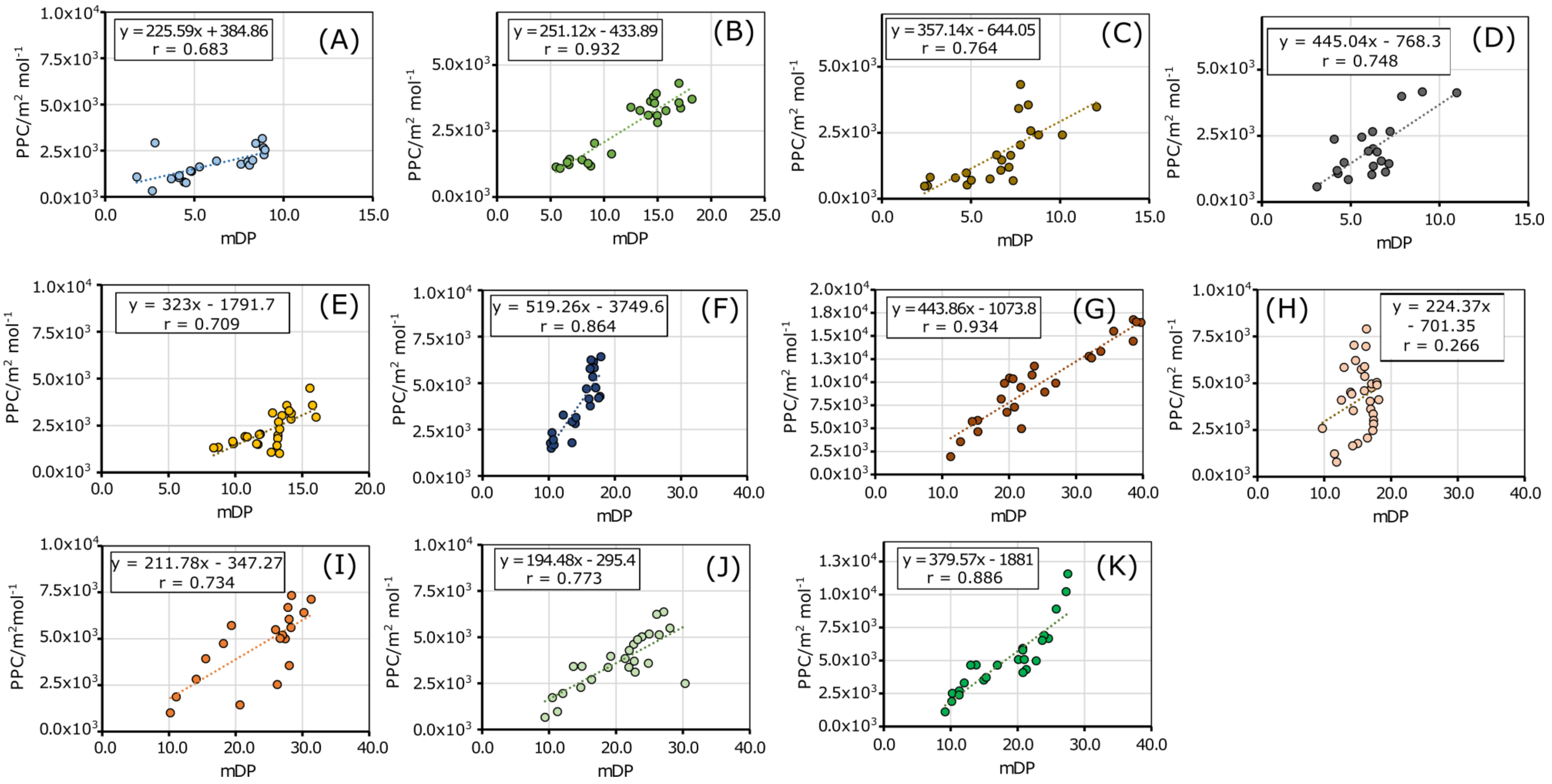

Figure S1. Protein precipitation capacity (PPC) as a function of mean degree of polymerization (mDP) of (A) *Aesculus hippocastanum*, (B) *Trifolium medium*, (C) *Rhododendron dichroanthum*, (D) *Rhododendron schlippenbachii*, (E) *Larix* sp, (F) *Lotus corniculatus*, (G) *Lysimachia vulgaris*, (H) *Pinus sylvestris*, (I) *Salix phylicofilia*, (J) *Ribes alpinum* and (K) *Trifolium repens*.

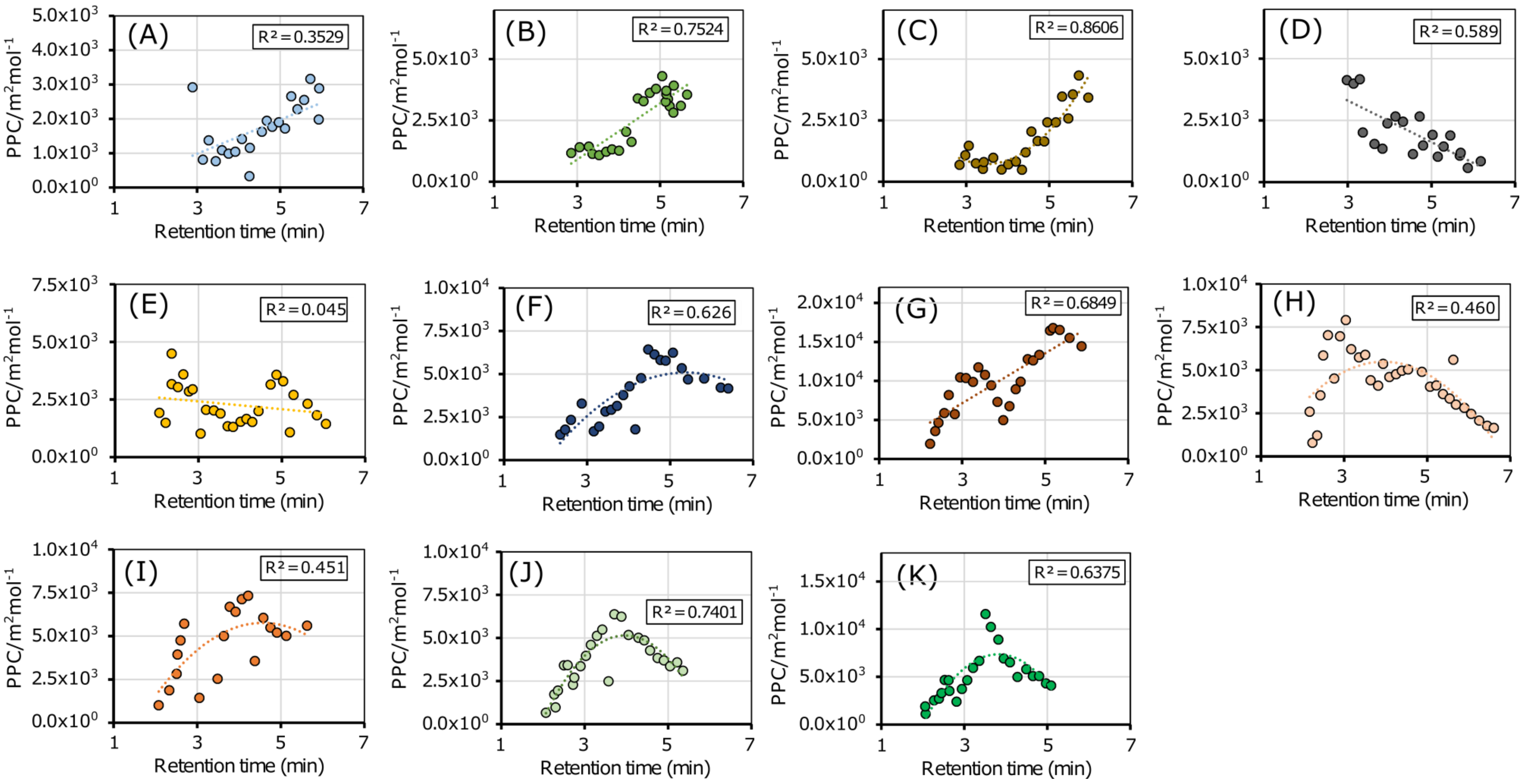

Figure S2. Protein precipitation capacity (PPC) as a function of retention time of (A) *Aesculus hippocastanum*, (B) *Trifolium medium*, (C) *Rhododendron dichroanthum*, (D) *Rhododendron schlippenbachii*, (E) *Larix* sp, (F) *Lotus corniculatus*, (G) *Lysimachia vulgaris*, (H) *Pinus sylvestris*, (I) *Salix phylicofilia*, (J) *Ribes alpinum* and (K) *Trifolium repens*.

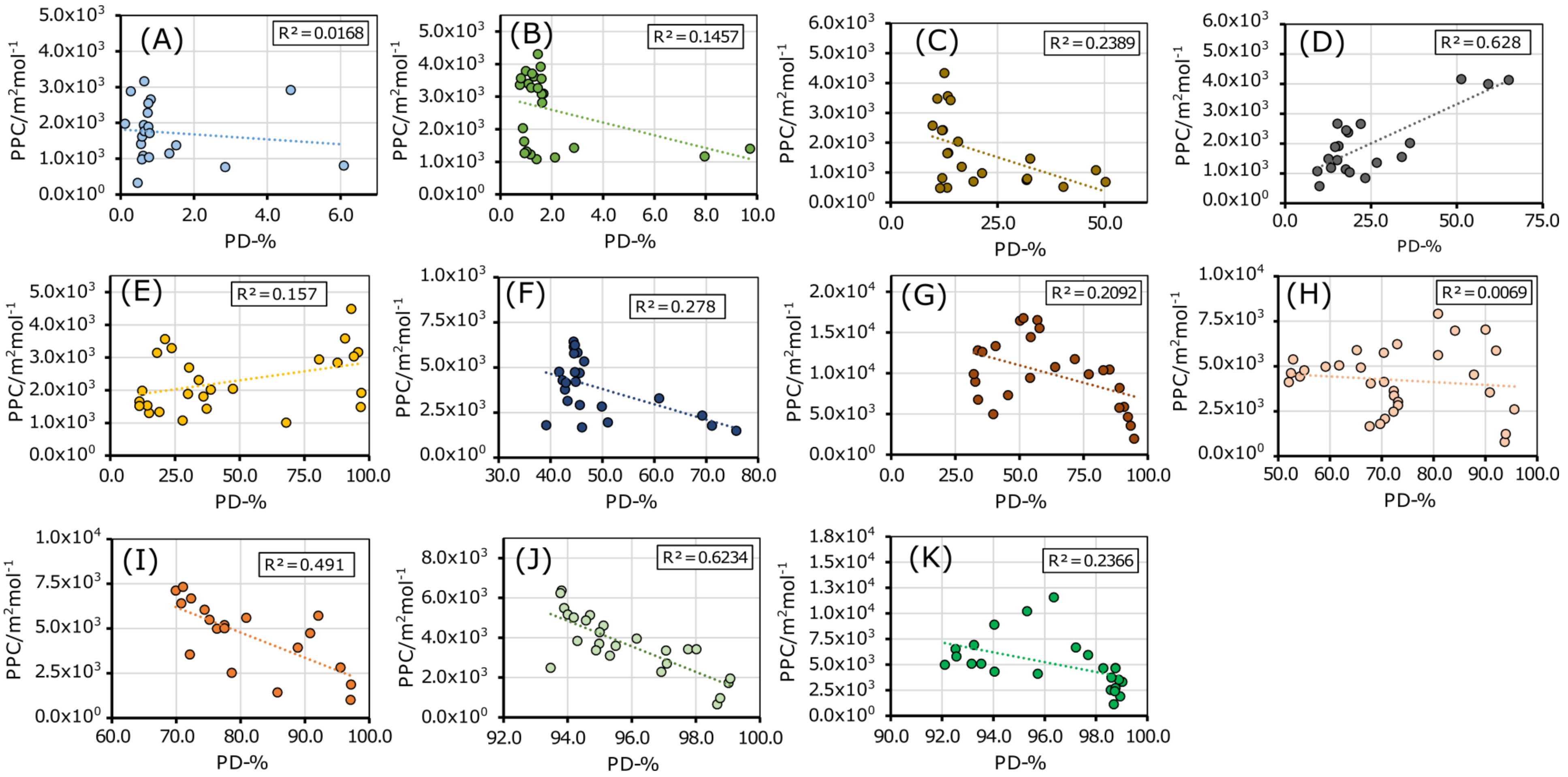

Figure S3. Protein precipitation capacity (PPC) as a function of prodelphinidin proportion (PD-%) of (A) *Aesculus hippocastanum*, (B) *Trifolium medium*, (C) *Rhododendron dichroanthum*, (D) *Rhododendron schlippenbachii*, (E) *Larix* sp, (F) *Lotus corniculatus*, (G) *Lysimachia vulgaris*, (H) *Pinus sylvestris*, (I) *Salix phylicofilia*, (J) *Ribes alpinum* and (K) *Trifolium repens*.

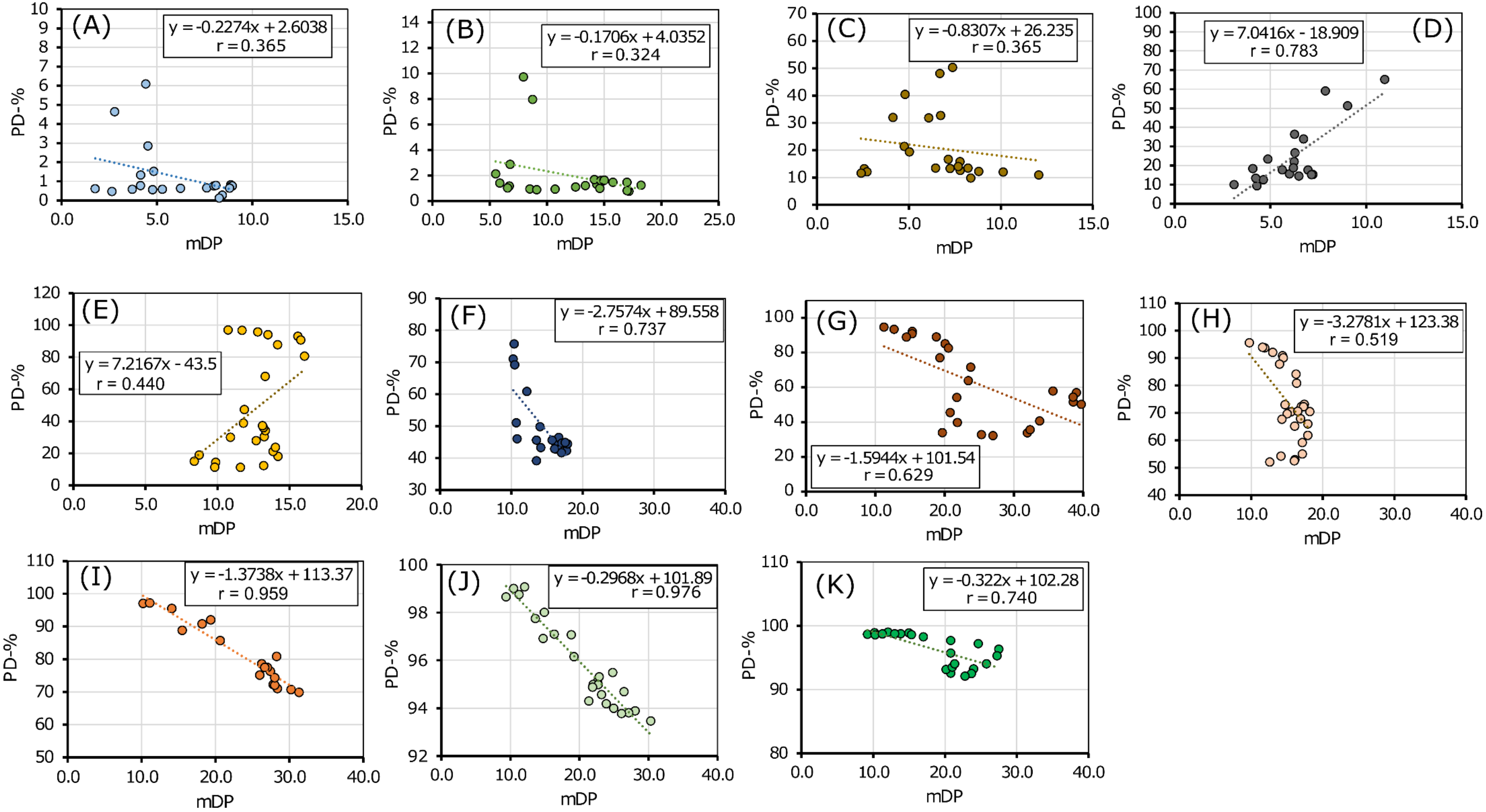

Figure S4 Prodelphinidin proportion (PD-%) as a function of the mean degree of polymerization (mDP) of (A) *Aesculus hippocastanum*, (B) *Trifolium medium*, (C) *Rhododendron dichroanthum*, (D) *Rhododendron schlippenbachii*, (E) *Larix* sp, (F) *Lotus corniculatus*, (G) *Lysimachia vulgaris*, (H) *Pinus sylvestris*, (I) *Salix phylicofilia*, (J) *Ribes alpinum* and (K) *Trifolium repens.*

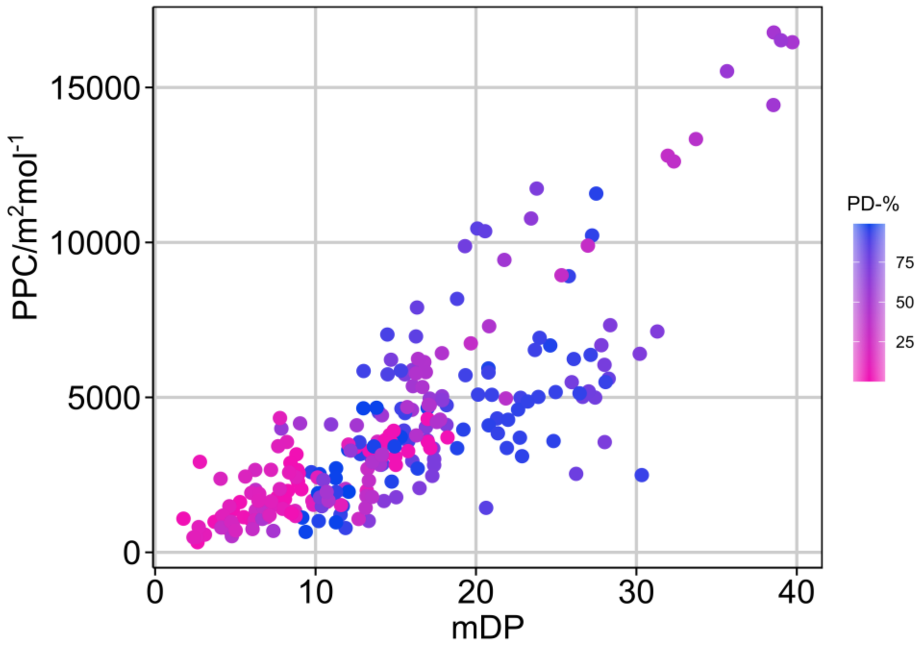

Figure S5. Protein precipitation capacity (PPC) as a function of the mDP. The colour gradient illustrates the PD-% of the fractions ranging from PC pure (pink) to PD pure (blue).

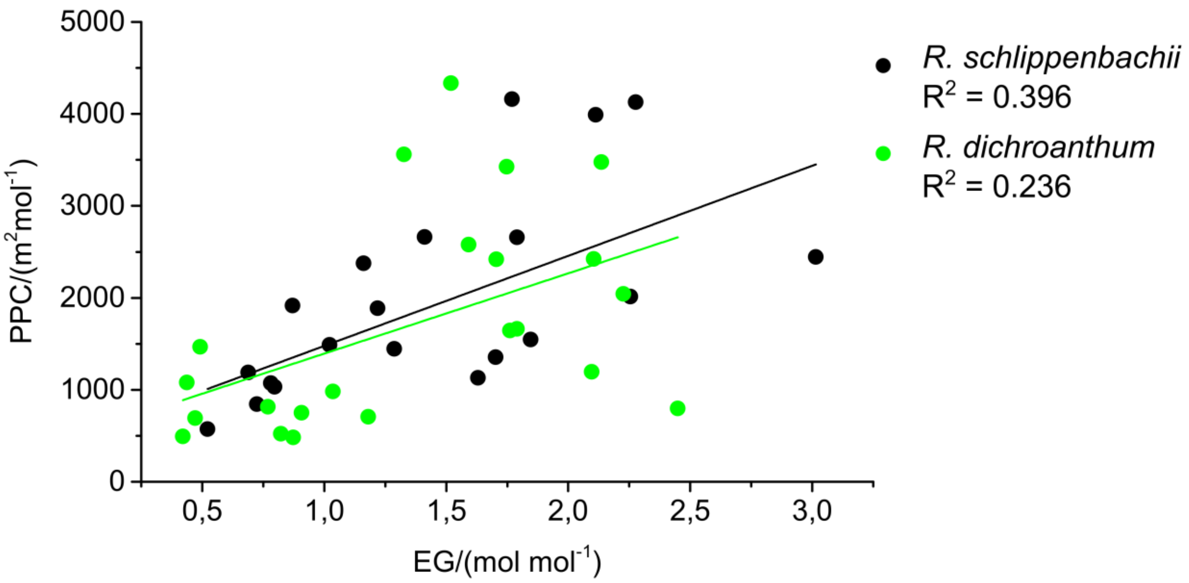

Figure S6. Protein precipitation capacity (PPC) as a function of the estimation of the relative galloyl content (EG).

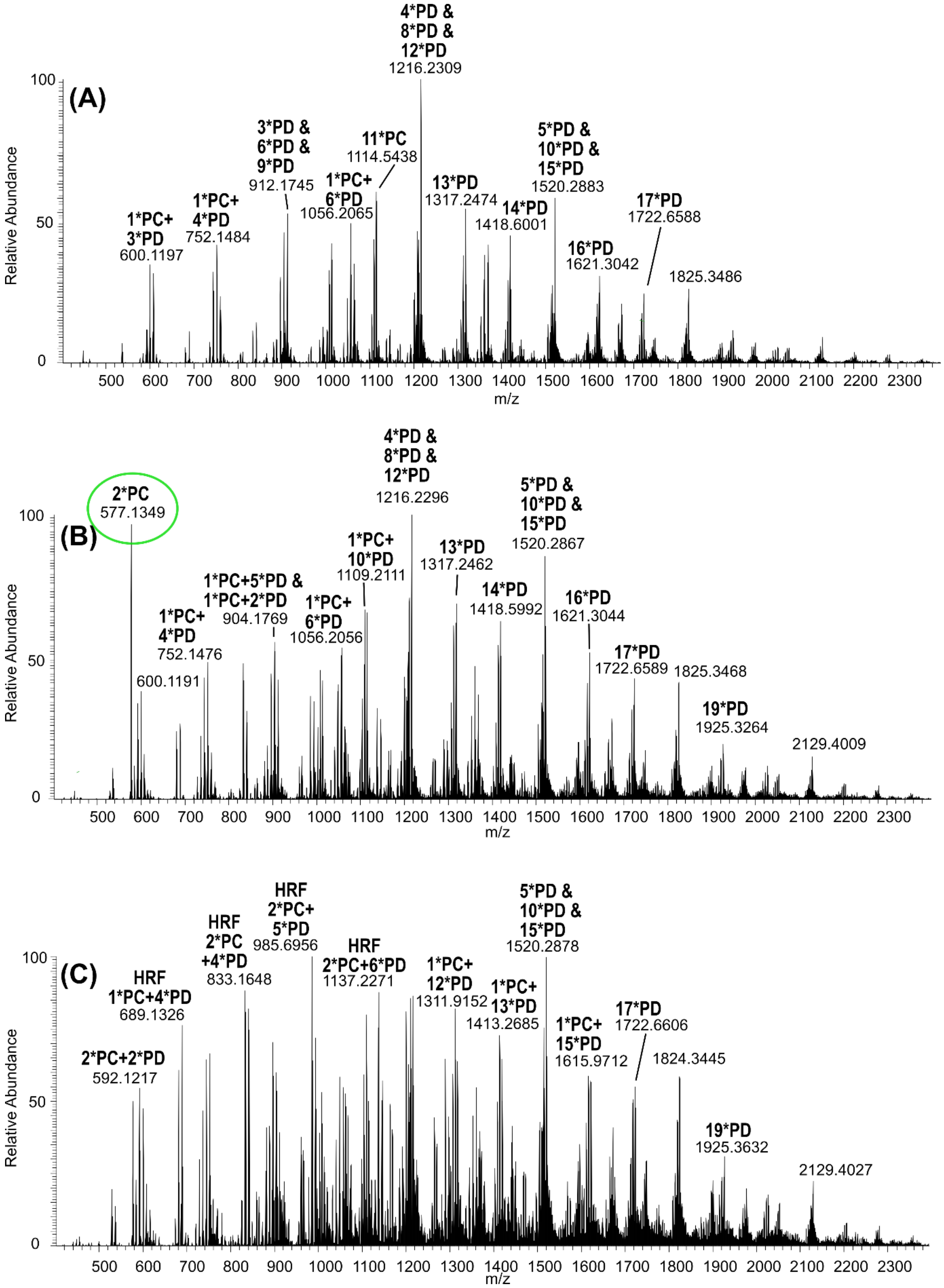

Figure S7. High-resolution mass spectra of *Ribes alpinum* semipreparative fraction numbers (A) 64, (B) 68 and (C) 72. Compound interpretations are presented above each signal. For instance "2*PC+2*PD" means a tetrameric proanthocyanidin consisting from two procyanidin units and two prodelphinidin units. All identified compounds were B-type proanthocyanidins consisting from procyanidins and prodelphinidins. The used abbreviations are as follows: PC = procyanidin, PD = prodelphinidin, HRF = heterocyclic ring fission.
